# Comparison of the Antioxidant Activities and Volatile Compounds of Coffee Beans Obtained Using Digestive Bio-Processing (Elephant Dung Coffee) and Commonly Known Processing Methods

**DOI:** 10.3390/antiox9050408

**Published:** 2020-05-11

**Authors:** Mesfin Haile, Hyung Min Bae, Won Hee Kang

**Affiliations:** 1 Department of Horticulture, Kangwon National University, Chuncheon 24341, Korea; mesfinhaile97@gmail.com (M.H.); dogpet2002@gmail.com (H.M.B.); 2 Convergence Program of Coffee Science, Kangwon National University, Chuncheon 24341, Korea

**Keywords:** antioxidants, elephant dung coffee, processing methods, volatile compounds

## Abstract

There are different types of coffee processing methods. The wet (WP) and dry processing (DP) methods are widely practiced in different parts of coffee-growing countries. There is also a digestive bioprocessing method in which the most expensive coffee is produced. The elephant dung coffee is produced using the digestive bioprocessing method. In the present experiment, the antioxidant activity and volatile compounds of coffee that have been processed using different methods were compared. The antioxidant activity, total phenolic content (TPC), total flavonoid content (TFC), and total tannin content (TTC) of green coffee beans from all treatments were higher as compared to roasted coffee beans. Regarding the green coffee beans, the 2,2-diphenyl-1-picrylhydrazyl (DPPH) radical scavenging activity of elephant dung coffee beans was higher as compared to that of the DP and WP coffee beans. The green coffee beans had higher DPPH activity and ferric reducing antioxidant power (FRAP) value compared to the roasted coffee beans. The green beans of elephant dung coffee had a high TPC than the beans obtained by WP and DP methods. TFC in elephant dung coffee in both green and roasted condition was improved in contrast to the beans processed using dry and wet methods. The elephant dung coffee had an increased TTC in comparison to the DP and WP coffee (green beans). About 37 volatile compounds of acids, alcohols, aldehydes, amide, esters, ethers, furans, furanones, ketones, phenols, pyrazines, pyridines, Heterocyclic N, and pyrroles functional classes have been found. Some of the most abundant volatile compounds detected in all treatments of coffee were 2-furanmethanol, acetic acid, 2-methylpyrazine, 2,6-dimethylpyrazine, pyridine, and 5-methylfurfural. Few volatile compounds have been detected only in elephant dung coffee. The principal component analysis (PCAs) was performed using the percentage of relative peak areas of the volatile compound classes and individual volatile compounds. This study will provide a better understanding of the impacts of processing methods on the antioxidants and volatile compounds of coffee.

## 1. Introduction

Coffee is an important crop in the world and widely known for its stimulant effect, health benefits, and economic importance. Post-harvest management activities have a prominent role in maintaining and enhancing the inherent qualities of coffee [1]. Processing methods applied to coffee have a significant contribution to improving the coffee flavor and aroma profile. There are three types of common coffee processing methods: wet-processing (WP), semi-dry processing, and dry-processing (DP). The WP method is widely used for processing Arabica coffee, except in some countries like Ethiopia, Yemen, and Brazil where both WP and DP methods are used [2]. The WP method requires adequate amounts of water and some special facilities for washing and fermentation processes [1]. The DP (natural) method produces coffee that is sweet, heavy in body, smooth, and complex. The DP method is used in the countries where water is scarce but where there is adequate sunshine for drying the coffee efficiently [2]. Moreover, there is also a unique coffee processing method called digestive bioprocessing. In this process, the coffee beans are passed through the digestive tracts of animals, for example, Kopi Luwak (civet coffee) and Black Ivory Coffee (elephant dung coffee) [3].

Civet coffee is one of the most expensive coffees in the world. It has a price tag of USD $500 per pound [4]. It is produced in Indonesia by feeding the fresh coffee cherries to the Asian palm civet (*Paradoxurus hermaphroditus*) to pass through its digestive tract [4]. The fresh coffee cherries are sweet, and the pulp is digested by the civet cat and the coffee beans are excreted in the feces. Different digestive enzymes, acids, and fermentation in the civet’s digestive tract add a unique flavor and aroma to the coffee beans. These flavors have been characterized as musty, earthy, smooth, syrupy, and chocolaty. Black Ivory Coffee (elephant dung coffee) is produced by Black Ivory Coffee Company in Thailand. It is produced by a method similar to that of producing civet coffee, except that the coffee cherries are eaten by elephants (*Elephas maximus).* The coffee beans pass through the elephant’s digestive tract in 12 to 70 h [5]. Individual parchment coffee beans are picked manually from the elephant’s excreta. Then the coffee is washed, dried, hulled, and sorted to choose the perfect green beans [6]. The Black Ivory Coffee has been characterized as having a very smooth taste as compared to regular coffee. It has a price tag of USD $1800 per kilogram, and its production is limited reaching approximately 200 kg per year [7].

The beneficial health effects of coffee are usually associated with its high antioxidant activity (ability to hinder the oxidation process). Several publications have compared the antioxidant properties in different popular drinks such as tea, coffee, and cocoa [8,9,10]. The antioxidant activity of plant extracts can be measured by several methods, including 2-diphenyl-1-picrylhydrazyl (DPPH) radical scavenging assay and ferric reducing antioxidant power (FRAP). The antioxidant activity of coffee is affected by the processing methods used to obtain green coffee beans. Recently, the green coffee beans got considerable attention in the pharmaceutical and nutraceutical industries because of their high antioxidant content [11]. Phenolic compounds are the main sources of antioxidant activity, and they also have other relevant biological and physicochemical properties [12]. The high level of antioxidant activity in green coffee beans might be associated with the total phenolic content (TPC) found in the beans [13]. Flavonoids are naturally present in plants. The growing research interest in flavonoids from dietary sources is due to the increasing evidence of the health benefits of flavonoids [14]. Flavonoids in food impart taste, color, and prevent fat oxidation [14]. Therefore, estimations of TPC, total flavonoids (TFC), and total tannin content (TTC) are important to evaluate the quality of coffee as well as any potential implications for health. 

The chemical composition of coffee is quite complex because coffee contains a wide range of non-volatile and volatile compounds that have diverse functionalities [15]. The green coffee beans are mostly comprised of carbohydrates, proteins, lipids, amino acids, peptides, organic acids, alkaloids, and phenolic compounds [16]. Several of these components are essential precursors of the coffee aroma during and after roasting [3,16,17,18]. During the roasting process, several physical and chemical phenomena occur inside the coffee beans, such as the Maillard reaction (the reaction between an amino acid and a reducing sugar), Strecker degradation (the reaction which converts α-amino acid into an aldehyde), and thermal degradation. Maillard reactions produced heterocyclic compounds, such as thiazoles, pyrazines, thiophenes, pyrroles, imidazoles, and pyridines [19]. Strecker degradation is a reaction between an amino acid and an α-dicarbonyl with the development of aminoketone that condenses to form nitrogen hetero-cyclic compounds or reacts with formaldehyde to form oxazoles [15]. During thermal degradation reactions, furans and aromatic compounds formed from a quinic acid moiety and a caffeic acid moiety of chlorogenic acid, respectively [20]. More than 900 volatile and semi-volatile compounds released during roasting, such as alcohols, acids, esters, aldehydes, ketones, furans, indoles, phenolic compounds, pyridines, pyrroles, thiols, and pyrazines [16,21,22,23]. These compounds impart the flavor and aroma to coffee [24]. Moreover, the process of preparing coffee, including the grinding and brewing techniques, also influence the aroma of brewed coffee [21,25]. The volatile compounds of coffee are efficiently identified using powerful analytical techniques such as gas chromatography–mass spectrometry (GC-MS) [5,21,22,26,27,28,29]. Therefore, in this study, the antioxidant activity and volatile compounds in coffee obtained by digestive bioprocessing, wet and dry processing methods have been compared.

## 2. Materials and Methods

### 2.1. Coffee Beans Samples Preparation and Chemicals

**Figure 1:**
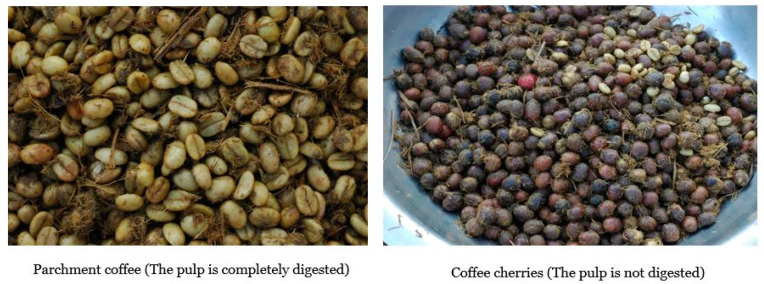
The elephant dung coffee right after the coffee cherry passed through the elephant digestive tract and collected from the elephant feces.

The coffee cherries were harvested from mature coffee trees (*Coffea arabica* L.) in Chandanpur, Nepal. Then, the harvested coffee cherries were divided into three groups to obtain raw coffee beans using three different types of coffee processing methods (digestive bioprocessing, wet and dry processing). The coffee cherries were eaten by elephants and excreted in two forms: one was the parchment coffee (the coffee pulps were completely digested) and the second was the coffee with its pulp (the pulp was not digested) (Figure 1). Like wet and dry processing methods, coffee obtained using the digestive bioprocessing method was subjected to the remaining steps like washing, drying, hulling and sorting. In total, there were four different treatments: WP (wet-processing), DP (dry-processing), and two groups of digestive bio-processed coffee: EP1 (the coffee pulp was completely digested) and EP2 (the pulp was not digested). The moisture content of coffee beans from all treatment was maintained at 11–12%. The coffee beans (600 g) were roasted at three different levels using an automatic roasting machine (GeneCafe CR-100 coffee roaster, Genesis, Ansan, Korea). These roasting levels were light roasting (245 °C for 11.5 min), medium roasting (245 °C for 13.5 min), and dark roasting (245 °C for 16 min).

Aluminum chloride (AlCl_3_·6H_2_O), sodium nitrite (NaNO_2_), sodium hydroxide (NaOH), monopotassium phosphate (KH_2_PO_4_), dipotassium phosphate (K_2_HPO_4_), and sodium carbonate (Na_2_CO_3_) were purchased from Dae-Jung Chemicals & Metals Co., Ltd., Gyeonggi-do, Korea. Folin-Ciocalteu’s phenol reagent, gallic acid, quercetin, 2,2-diphenyl-1-picrylhydrazyl, trichloroacetic acid (C_2_HC_l3_O_2_), potassium ferricyanide (C_6_N_6_FeK_3_), ferric chloride (FeCl_3_), and C8–C40 alkanes (40 mg/L each, in hexane) were purchased from Sigma Aldrich LLC (St. Louis, MO, USA). Methanol was supplied by Merck KGaA (Darmstadt, Germany).

### 2.2. Preparation of Coffee Extracts for Antioxidant Activity Assay

The green and roasted coffee beans (10 g) were ground from each treatment. Then, 2 g of coffee powder sample from each treatment was measured and mixed with 20 mL of deionized distilled water (ddH_2_O). Each sample was sonicated for 30 min (70% amplitude, 0.7 s cycle) and then mixed again by using a magnetic stirrer for a further 30 min under moderate heat (35 °C) to increase the extraction yield. The coffee extract of each sample was centrifuged (7000× *g*, 10 min, 25 °C) to separate the solid residue. Finally, these extracts were used to analyze the antioxidant activity, total phenol content (TPC), total flavonoid content (TFC) and total tannin content (TTC).

### 2.3. Colorimeter Data of Roasted Coffee

The color reading was estimated using a Chroma Meter (CR-400 Chroma Meter, Tokyo, Japan). A small amount of coffee powder (5 g) was placed in a Petri dish to record a* (redness), b*(yellowness), and L* (lightness). The color parameters reading were made in triplicate. 

### 2.4. Antioxidants Activity Assays

#### 2.4.1. 2,2-Diphenyl-1-picrylhydrazyl (DPPH) Radical Scavenging Assay

The DPPH activity was measured using the protocol described by Pataro et al. [30], with some modifications. The DPPH solution (0.1 mM) was prepared using methanol. The sample reaction was prepared by mixing 800 μL coffee extract with 3.2 mL methanol-DPPH solution. The control reaction was prepared by mixing 800 μL ddH_2_O with 3.2 mL methanol-DPPH solution. The absorbance of the samples and the control solution was recorded using a spectrophotometer (U-2900, Hitachi High-Tech Corporation, Tokyo, Japan) at 515 nm after 30 min of incubation in a dark condition. Methanol was used as a blank. The assay was done in triplicate. The DPPH inhibition percentage was calculated using the following formula:

DPPH inhibition (%) = (A_control_ − A_sample_)/A_control_ × 100 (1)

where A_control_ is the absorbance of control reaction (DPPH and Methanol) and A_sample_ is the absorbance in the presence of coffee extract.

#### 2.4.2. Ferric Reducing Antioxidant Power (FRAP)

FRAP assay was conducted using the protocol described by Oyaizu [31]. Coffee extract (2.5 mL) was mixed with 2.5 mL of 200 mM sodium phosphate buffer (pH 6.6) and 2.5 mL of 1% potassium ferricyanide. The mixture was incubated for 20 min at 50 °C. Then, 2.5 mL of 10% trichloroacetic acid was added. The mixture was centrifuged (2000× *g* for 10 min) and 5 mL of the supernatant mixed with another 5 mL of ddH_2_O and 1 mL of 0.1% ferric chloride. The absorbance reading was taken at 700 nm using a spectrophotometer (U-2900, Hitachi High-Tech Corporation, Tokyo, Japan). Ascorbic acid was used as standard. The FRAP assay was done in three replications. 

### 2.5. Total Phenolic Content (TPC)

TPC of the coffee extracts was determined using Folin-Ciocalteu reagent, as previously described by Singleton et al. [32], with some modifications. The coffee extract (20 μL) was diluted with 1580 μL ddH_2_O. Diluted coffee extract (160 μL) and Ciocalteu’s phenol reagent (10 μL) were mixed and held for 8 min. Then, 30 μL of 20% Na_2_CO_3_ solution was mixed and incubated in dark condition for 2 h. The absorbance readings were made at 765 nm using a UV/visible spectrophotometer (U-2900, Hitachi High-TechCorporation, Tokyo, Japan) against a blank containing all reagents and replaced the coffee extract by ddH_2_O. Gallic acid solutions (0–1 mg/mL) were used to generate a standard curve (*r^2^* = 0.997). The estimation of the TPC was executed in triplicate. Results were expressed as mg gallic acid equivalent/mL (mg GAE/mL) of coffee extract.

### 2.6. Total Flavonoid Content (TFC)

TFC of each coffee extract was measured according to the protocol stated by Dewanto et al. [33], with some modifications. Coffee extract (250 μL), ddH_2_O (1 mL), and 75 μL of 5% NaNO_2_ were mixed together. After 5 min, 10% AlCl_3_·6H_2_O solution (150 μL) was added and incubated for 6 min. We added 1 N NaOH (500 μL), and the mixture was incubated for 11 min. Instead of coffee extract, the ddH_2_O was replaced and used as a blank. The absorbance of the sample was estimated at 510 nm using a UV/visible spectrophotometer (U-2900, Hitachi High-Tech Corporation, Tokyo, Japan). The standard solution was prepared using a quercetin solution (0–1 mg/mL). The total flavonoids in the coffee were expressed as mg quercetin equivalent/mL of coffee extract. TFC determination was done in triplicate.

### 2.7. Total Tannin Content (TTC)

Total tannin content (TTC) was estimated using the Folin–Ciocalteu method, with some modifications [34]. Coffee extract (100 μL) was added to a 10 mL tube containing ddH_2_O (7500 μL), 500 μL of Folin-Ciocalteu phenol reagent, and 1000 μL of 35% sodium carbonate solution. The mixture was well mixed and incubated at room temperature for 30 min. A set of standard solutions of tannic acid (20, 40, 60, 80, 100 μg/mL) was made as a reference. Absorbance for the test and standard solutions was measured with a UV/Visible spectrophotometer (U-2900, Hitachi High-Tech Corporation, Tokyo, Japan) at 700 nm. Distilled water was used as a blank. The tannin content was expressed in terms of mg/mL of tannic acid in the coffee extracts. The determination of the TTC was done in triplicate.

### 2.8. Analysis of Volatile Compounds

The medium-level roasted coffee beans have a mild taste and are widely supplied by a roasting company in Korea. Therefore, the medium-roasted coffee beans were chosen and ground into a fine powder (200–400 μm) using a grinder (Latina 600N electric grinder). The coffee powder was weighed (10 g) to obtain 35 mL of extracts using an espresso machine (Model: MINI-CONTROL 1 GR. C/M, CREM A.C, S.L.). This method involved the extraction of compounds from ground coffee using high pressure (15 bar) and hot water (91–95 °C). Immediately after extraction, 1 mL of the brewed coffee sample from each treatment was transferred into a 10 mL headspace vial with a screw top and tightly closed. The preparation of each coffee brew sample was made in duplicate. 

The volatile compounds of coffee were analyzed using GC coupled to a mass spectrometer detector (GC–MS, 7890A–5975C; Agilent Technologies, Inc., Santa Clara, CA, USA), as described by Thammarat et al. [5], with some modification. The extraction temperature was set at 80 °C with an equilibration time of 60 min. The vials were shaken at the maximum frequency of 250 times/min. The vial pressurization and injection time were set at 15 psi and 0.5 min, respectively. The sampled headspace containing volatile compounds was directly introduced into a GC-MS system. The GC injector temperature and split ratio were set at 230 °C and 5:1, respectively. The volatile compounds were separated on a VF-WAXms column (30 m × 0.25mm, 0.2 µm film thickness. Helium was used as a carrier gas, with a constant flow rate (1 mL/min). The oven temperature program was as follows: initial temperature of 40 °C, heated to 200 °C at a rate of 3 °C /min, increased to 230 °C at a rate of 50 °C/min, and then held for 2 min. The ion source temperature was set at 230 °C. The magnitude of the electron ionization (EI) voltage was 70 eV. Mass spectra were acquired over a scan range of 35 to 300 m/z. The standards (C8–C40, alkanes: 40 mg/L each, in hexane) were prepared in different concentrations (0, 25, 50, and 100 μg/L), and the calibration curve (*r^2^* = 0.99) was generated to quantify the volatile compounds in the brewed coffee samples. The samples were analyzed in duplicate.

### 2.9. Statistical Analysis

Analysis of variance (ANOVA) was computed for testing the significances of the experiment. Data were summarized in an Excel program and analyzed using SAS 9.4 software (SAS Institute, Cary, NC, USA). Mean separation was done using Fisher’s Least Significant Difference. The principal component analysis (PCA) of volatile compounds was analyzed and summarized using XLSTA version 2015.1 (Addinsoft Inc., 244 Fifth Avenue, Suite E100, New York, NY, USA).

## 3. Results and Discussion

### 3.1. Colorimeter Data of Roasted Coffee

The color of the coffee beans resulting from roasting is commonly used to describe the level of roasting, and the color ranges from light to dark. The coffee color, aroma, and flavor profiles are developed during roasting. It is important to decide the right roasting condition according to the type of coffee being roasted. Controlling the roasting time and temperature are important to develop the required chemical reaction without burning the coffee beans and to preserve the flavors in the brewed coffee [35]. At all roasting levels, there was a significant difference (*p* < 0.05) between EP1 and EP2 coffee beans regarding L* and b* values. However, a* values between these two treatments did not significantly differ (*p* > 0.05) (Table 1). The EP2 coffee beans showed a significantly (*p* < 0.05) higher L* and b* values compared to other treatments at all roasting levels. The DP coffee beans had lower L* value compared to other treatments at light and medium roasting conditions. The WP coffee beans at medium roasting conditions had higher L* value compared to DP and EP1 coffee beans. This inconsistency might be associated with the varied response of coffee beans to the roasting time and temperature. Variations in color parameters are because of the methods of processing applied to obtain the raw coffee beans and roasting temperature. The color of the green coffee beans was affected by the type of coffee processing methods implemented and its impact was also noted in the color of roasted coffee. Yellow-green raw beans turn into brown-black color after roasting [36]. As the roasting time extended from 11 to 16 min (light to dark roasting), the color of the coffee beans turned from light brown to dark black and results in the reduction of L* a*, and b* values (Table 1). The change in the color of the coffee beans during roasting might be associated with the non-enzymatic browning (caramelization) and pyrolysis reaction.

### 3.2. Effect of Processing Methods on the Antioxidant Activities of Coffee

**Figure 2:**
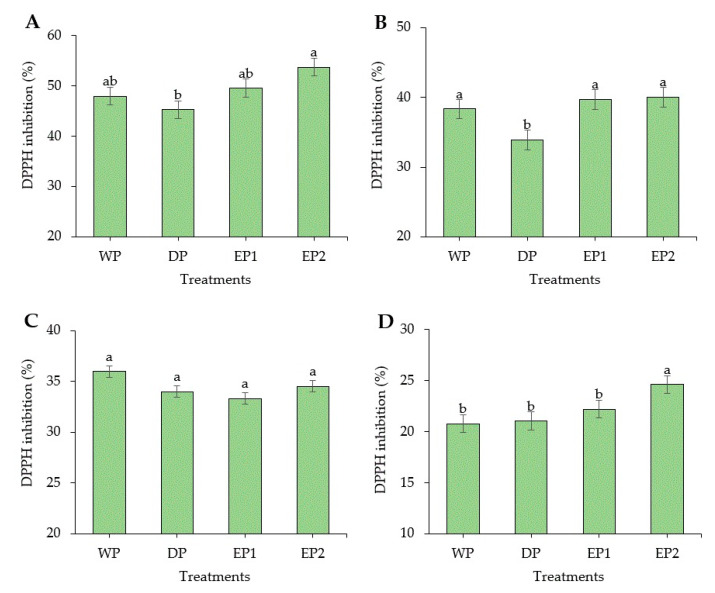
2,2-diphenyl-1-picrylhydrazyl (DPPH) inhibition (%) of green and roasted coffee beans obtained using different processing methods. ((**A**): Green beans; (**B**): Light roasted, (**C**): Medium roasted; (**D**): Dark roasted). EP1: elephant dung coffee (pulp was completely digested); EP2: elephant dung coffee (pulp was not digested); WP: wet-processed beans; DP: dry-processed beans. Different letters above the bars indicate statistically significant difference at *p* < 0.05.

**Figure 3:**
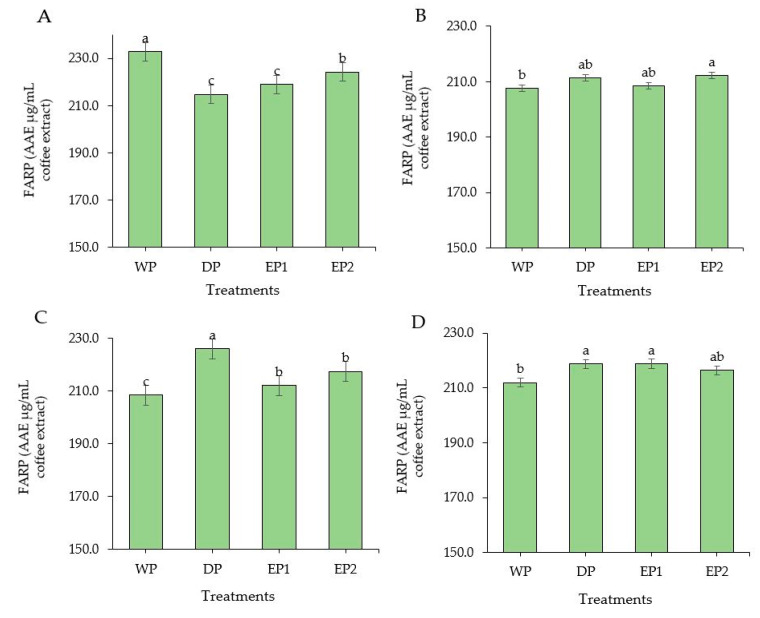
Ferric-reducing antioxidant power (FRAP) of green and roasted coffee beans that were processed with different methods. ((**A**): Green beans; (**B**): Light roasted, (**C**): Medium roasted; (**D**): Dark roasted). EP1: elephant dung coffee (pulp was completely digested); EP2: elephant dung coffee (pulp was not digested); WP: wet-processed beans; DP: dry-processed beans. Different letters above the bars indicate statistically significant difference at *p* < 0.05.

Coffee is considered as a rich source of antioxidants. In some countries, coffee is the source of antioxidants for nearly two-thirds of the population [37]. Regarding the green coffee beans, the DPPH radical scavenging activity of elephant dung coffee beans (EP1 and EP2) was higher compared to the DP and WP coffee beans (Figure 2A). However, the FRAP was higher in green coffee beans obtained by the WP (232.74 AAEµg/mL of coffee extract) compared to others (Figure 3A). The EP2 treatment of green beans had significantly higher (*p* < 0.05) DPPH inhibition percentage (53.68%) compared to the coffee beans processed using the DP method. The increased antioxidant activity might be associated with the increased TPC of coffee beans. This result is supported by Naidu et al. [13], who reported that the increased DPPH activity in green coffee had a relation with the improved TPC content. The FRAP value of elephant dung coffee beans (green) was 218.92 and 224.22 AAEµg/mL of coffee extract in EP1 and EP2 treatments, respectively (Figure 3A). The change in the antioxidant properties of coffee is because of the degradation of the commonly existing antioxidants and the production of the new ones during the process. The antioxidant activity of coffee depends on the availability of both naturally existing constituent and the compounds produced during processing [38]. Under the light roasting condition, the DPPH inhibition percentage was higher in EP1 (39.74%) and EP2 (40.04%) treatment (Figure 2B). Similarly, the FRAP was significantly higher (*p* < 0.05) in EP2 (212.2 AAEµg/mL of coffee extract) coffee treated to light roasting as compared to coffee beans obtained using the WP (Figure 3B). However, the result differed with each roasting condition. Under medium-roasting, EP1 and EP2 had lower FRAP compared to beans obtained using the DP method (Figure 3C). In medium-roasted coffee beans, the DPPH activity did not significantly differ among treatments (Figure 2C). Under the dark-roasting condition, the DPPH radical scavenging activity was significantly higher (*p* < 0.05) in EP2 (24.61%) (Figure 2D), and the FRAP was higher in EP1 (Figure 3D). Generally, the DPPH activity dropped as the roasting condition changed from light to dark. This result coincides with the results obtained by Cho et al. [39], who reported that the lighter roasted coffee showed a higher antioxidant activity as compared to dark roasted coffee and green coffee beans. The origin, harvesting, post-harvest processing, and style of preparing the coffee beverage are the factors that greatly influence the total antioxidant activity of coffee [40].

The green coffee beans had higher DPPH and FRAP activity compared to the roasted coffee beans. The response of coffee varieties varies with different roasting time and temperature and affects the antioxidant activities. Several reports mentioned that the roasted beans showed more antioxidant activity than the green bean. Priftis et al. [41] studied and compared the antioxidant activity of green and roasted coffee beans in 13 coffee varieties (12 from *Coffea arabica* L. and 1 from *Coffea canephora* species). They mentioned that the DPPH values of roasted beans were higher than those of green beans in eight coffee varieties. However, they also reported a result that is in agreement with the present work. Among the tasted coffee varieties, five of them (four from *Coffea arabica* L. and the *Coffea canephora* species) responded differently, and the green coffee beans showed higher DPPH activity than the roasted coffee beans.

### 3.3. Total Phenolic Content

**Figure 4:**
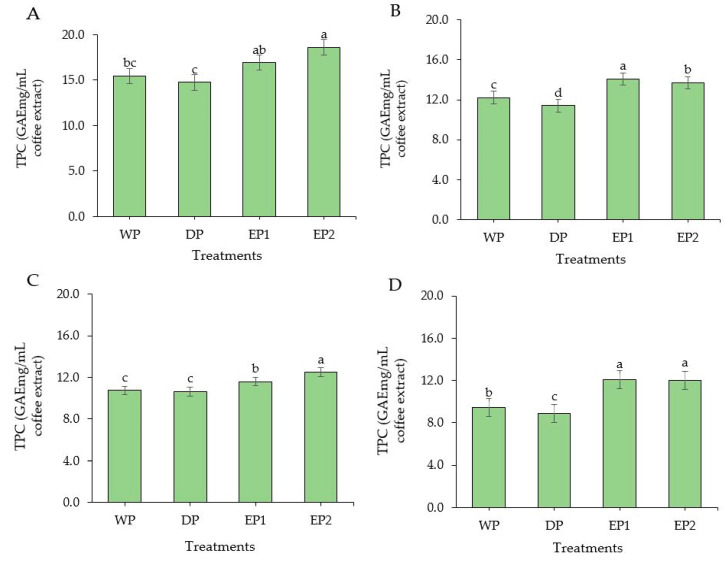
Total phenolic content of green and roasted coffee beans that obtained using different processing methods. ((**A**): Green beans; (**B**): Light roasted, (**C**): Medium roasted; (**D**): Dark roasted). EP1: elephant dung coffee (pulp was completely digested); EP2: elephant dung coffee (pulp was not digested); WP: wet-processed beans; DP: dry-processed beans. Different letters above the bars indicate statistically significant difference at *p* < 0.05.

The TPC was found to be significantly higher (*p* < 0.05) in EP1 and EP2 coffee (green beans) compared to the coffee beans obtained by WP and DP methods (Figure 4A). The TPC of EP1 and EP2 coffee (green beans) was 16.92 and 18.59 GAEmg/mL of coffee extract, respectively (Figure 4A). The TPC was found in a significantly greater (*p* < 0.05) amount in both EP1 and EP2 coffee beans subjected to light, medium, and dark roasting levels as compared to coffee beans obtained from WP and DP (Figure 4B–D). This increased TPC in EP1 and EP2 might be associated with the processing methods. As the coffee beans passed through the elephant’s digestive tract, the coffee beans were exposed to various acids, alcohols, and digestive enzymes that may have contributed to the degradation of the complex molecules of coffee beans and increased the phenolic contents during the extraction time. Under the light roasting condition, the TPC of EP1 and EP2 coffee beans was 14.08 and 13.70 GAEmg/mL of coffee extract, respectively. Relatively, the coffee beans obtained by the DP method had lower TPC compared to the WP method. In the WP method, the coffee cherries undergo a fermentation process which removes the mucilage layer and leave cleaned parchment coffee. The various bacteria and yeasts that cause a spontaneous fermentation also produce different acids, alcohols, and enzymes and greatly improves the antioxidant and phenolic content of coffee. Regarding the roasting condition, the highest TPC was obtained in lightly roasted EP1 and EP2 coffee beans. This agrees with the findings of Bita and Preda [42]. They roasted the coffee beans at three temperatures 200 °C, 220 °C, and 240 °C and found that TPC decreased with increasing temperature. In general, the green coffee beans had higher TPC from all treatments compared to roasted beans. This result agrees with that of Odžaković et al. [43], who reported that TPC was significantly higher (*p* < 0.05) in green coffee beans compared to TPC in roasted coffee, and TPC decreases as the roasting temperature increases.

### 3.4. Total Flavonoid Content

**Figure 5:**
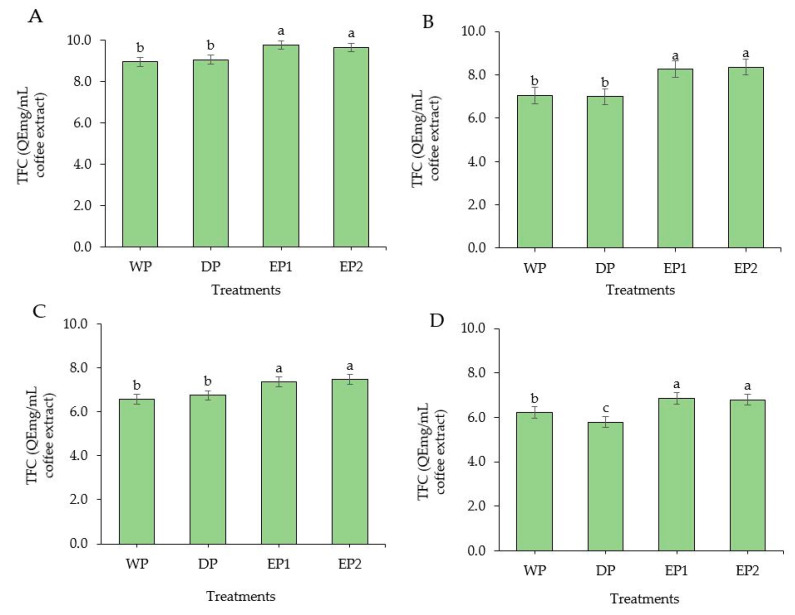
Total flavonoid content of green and roasted coffee beans that processed with different methods. ((**A**): Green beans; (**B**): Light roasted, (**C**): Medium roasted; (**D**): Dark roasted). EP1: elephant dung coffee (pulp was completely digested); EP2: elephant dung coffee (pulp was not digested); WP: wet-processed beans; DP: dry-processed beans. Different letters above the bars indicate statistically significant difference at *p* < 0.05.

TFC was improved significantly in EP1 and EP2 coffee beans (green and roasted) as compared to the coffee beans obtained from WP and DP methods (Figure 5A–D). The TFC of green coffee beans was 9.79 and 9.66 QEmg/mL of coffee extract in EP1 and EP2, respectively (Figure 5A). There was no significant difference (*p* > 0.05) between WP and DP processed coffee beans (green and roasted beans), except at the dark roasting level, at which the WP coffee beans had a significantly higher (*p* < 0.05) TFC compared to DP coffee beans. The TFC of lightly roasted coffee beans was 7.05, 6.99, 8.27, and 8.36 QEmg/mL of coffee extract in WP, DP, EP1, and EP2 treatments, respectively (Figure 5B). In general, the green beans under all treatments had higher TFC compared to the respective roasted beans. This result accords with Hudakova et al. [44], who reported the highest flavonoids content in unroasted coffee Arabica beans. In addition, referring to the roasting levels, lightly roasted coffee yielded a higher TFC than the medium and dark roasted coffee from all treatments. In a study conducted by Lee et al. [45], it was observed that the coffee sample roasted at the lowest temperature showed the highest TFC. Otherwise, previously obtained results by Hecimovic et al. [46] and Odžaković et al. [43] proved that the highest flavonoid content was in dark roasted coffee beans, whereas the other coffee varieties had more flavonoid in light and medium roasted coffee. These various results from several past studies may have been the result of the origins, varieties, and processing methods.

### 3.5. Total Tannin Content

**Figure 6:**
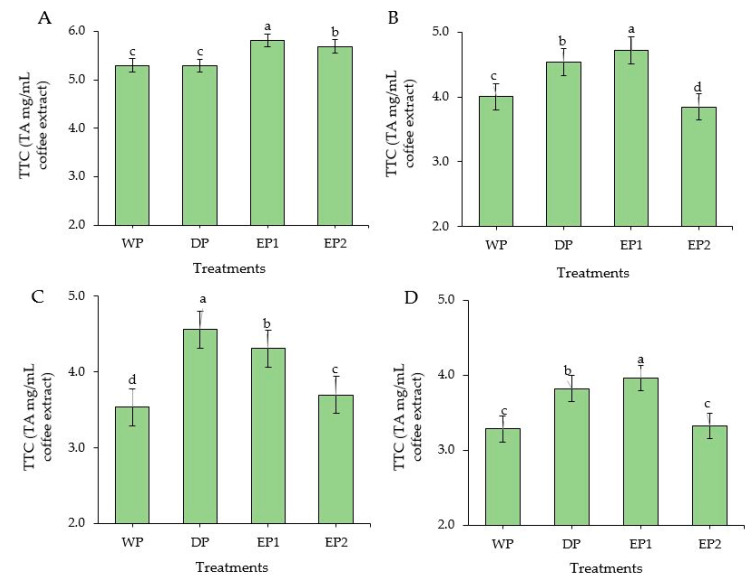
Total tannin content of green and roasted coffee beans that obtained using different processing methods. ((**A**): Green beans; (**B**): Light roasted, (**C**): Medium roasted; (**D**): Dark roasted). EP1: elephant dung coffee (pulp was completely digested); EP2: elephant dung coffee (pulp was not digested); WP: wet-processed beans; DP: dry-processed beans. Different letters above the bars indicate statistically significant difference at *p* < 0.05.

The EP1 and EP2 green coffee beans had significantly higher (*p* < 0.05) TTC compared to DP and WP green coffee beans. TTC of EP1 and EP2 green coffee beans was 5.81 and 5.29 TAEmg/mL of coffee extract, respectively (Figure 6A). During the light roasting level, the TTC of EP2 was significantly lower and followed by WP coffee beans. The TTC was significantly higher (*p* < 0.05) in EP1 treatment both in green and roasted (light and dark level) coffee (Figure 6B,C). The DP treatment yielded significantly higher (*p* < 0.05) TTC in the medium roasted beans, followed by EP1 beans. Otherwise, with medium roasting, TTC was notably reduced in WP and EP2 beans. At dark roasting levels, the EP2 coffee beans (3.32 TAEmg/mL) had a lower TTC than EP1 (3.96 TAEmg/mL) and DP coffee beans (3.82 TAEmg/mL of coffee extract) (Figure 6D). Relatively, the TTC was found in higher amounts in green beans than in roasted beans obtained from all treatments. Regarding the roasting conditions, the coffee beans obtained from all treatments produced a higher TTC in light roasting condition, except for DP beans. This result is in agreement with Hecimovic et al. [46], who found the highest TTC in a *C. arabica* variety (Minas) at the light roasting level, while the other variety (Ciccolatato) showed the highest TTC at the medium roasting level. For the most part, as the roasting temperature and time increased, the TTC content decreased in coffee [47], as it decreases also in soya bean flour [48] and maize [49].

### 3.6. Effect of Processing Methods on Volatile Compounds of Coffee

**Figure 7:**
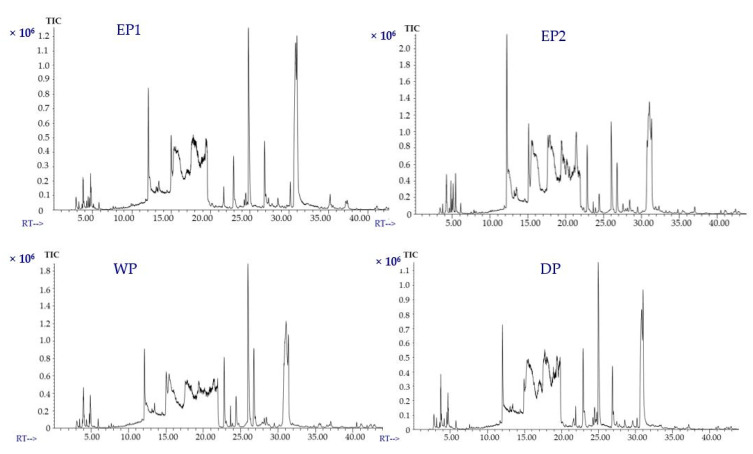
Total ion chromatograms (TICs) of volatile compounds of coffee (brewed samples). (EP1: elephant dung coffee (pulp was completely digested); EP2: elephant dung coffee (pulp was not digested); WP: wet-processed coffee beans; DP: dry-processed coffee beans). RT: Retention time (minutes).

The volatile compounds of coffee treated with various processing methods were identified using static headspace gas chromatography–mass spectrometry (SHS-GC/MS). The volatile compounds in coffee are either naturally present in the green beans or develop during the post-harvest operations (mucilage removal process, drying, and storage) [50]. Green coffee beans are characterized by an unpleasant taste. The development of flavor is achieved through thermal reactions during roasting and brewing. The volatile compounds of green coffee beans were found to have a notable impact on the aromatic properties of roasted coffee beans [51]. The total ion chromatograms of the EP1 and EP2 and control (WP and DP) samples are presented in Figure 7. Volatile profiles of coffee beans obtained from different processing methods were compared for about 37 selected compounds that were acids, alcohols, aldehydes, amide, esters, ethers, furans, furanones, ketones, phenols, pyrazines, pyridines, Heterocyclic N and of pyrroles functional classes. The concentrations of volatile compounds in mg/L of the coffee extract are represented in Table 2; Table 3. One of the most abundant volatile compounds found in all coffees from various processing treatments is 2-furanmethanol. It was found in higher amounts in EP2 coffee beans (8.12 mg/L of coffee extract) and WP coffee beans (7.54 mg/L of coffee extract) than in EP1 and DP coffee treatments. This result agrees with the findings of Ifmalinda et al. [52], who identified the volatile compounds in civet coffee (Luwak coffee) and found 2-furanmethanol to be in a higher amount than in the control sample (Arabica coffee). The amount of 2-furanmethanol was 4.76, and 4.11 mg/L of coffee extract from EP1 and DP beans, respectively.

The second most abundant compound was acetic acid. Its concentration in EP1, EP2, WP, and DP beans was 2.38, 3.60, 4.17, and 3.10 mg/L of coffee extract, respectively (Table 2). Relatively, the maximum levels of acetic acid were found in DP and EP2 coffees, respectively. These variations in the concentration of volatile compounds might be related to the processing methods used for obtaining the raw coffee beans. As Rodrigues et al. [53] reported, the implemented processing methods have a direct influence on the quality of the final coffee beverage. The amounts of volatile compounds of coffee vary according to coffee species and varieties. They also mention that these two compounds (furan methanol and acetic acid) were found in higher amounts in Arabica coffee than in Robusta [53]. The furan methanol and acetic acid compounds have been reported as good indicators to distinguish among coffees roasted at different levels from light to dark [53]. One of the most important aromatic compounds in coffee is 2-methylpyrazine. Its aroma is described as nutty, roasted, chocolate, and cocoa. The concentration of 2-methylpyrazine was lower in EP1 and DP coffee samples than in WP and EP2. The 2-methylpyrazine concentration in brewed coffee samples was 3.86, 3.10, 2.74, and 1.20 mg/L in WP, EP2, DP, and EP1, respectively (Table 2). An important contributor to the aroma and taste of different foods including coffee and wines is 2,6-dimethylpyrazine. It is found in cereals and cereal products. It has also been used as an odorant and flavor additive in foods. Its aroma is described as nutty and coffee-like. The EP2 beans had a relatively higher concentration of 2,6-dimethylpyrazine than other coffee bean samples. The WP beans had a lower concentration of 2,6-dimethylpyrazine. The average concentrations of 2,6-dimethylpyrazine in coffee brewed from EP2, EP1, WP, and DP beans were 2.83, 1.20, 0.92, and 1.48 mg/L, respectively.

Caporaso et al. [54], described that pyridine is a volatile compound found in coffee and is mostly produced during roasting as a decomposition product of trigonelline. The pyridine content of EP2 coffee (3.26 mg/L of brewed coffee) was higher, almost twice that of coffee beans from other treatments. The amount of pyridine was 1.33, 1.75, and 1.74 mg/L of brewed coffee from EP1, WP, and DP beans, respectively. Pyridine has a fishy odor. Vegetables (such as asparagus), fruit (apples), and meat (fatty fish, cooked beef, and raw chicken) contain 5-Methylfurfural. It is known for its caramelly aroma. The amount of 5-Methylfurfural was found to be higher in WP beans (2.41 mg/L of brewed coffee) and twice as much as in coffee obtained by other treatments. The DP coffee had a low concentration of 5-methylfurfural, that is, 0.97 mg/L of brewed coffee (Table 2). The WP coffee (2.01 mg/L) had a higher amount of furfural content, twice as much as in the brewed coffee of EP1 (0.87 mg/L) and DP (1.08 mg/L). The concentration of furfural compound in EP2 coffee was 1.31 mg/L of brewed coffee. The concentration of 2-methylbutanal in EP1 and DP coffee was 0.52 mg/L and 0.43 mg/L of brewed coffee, respectively. The 2-methylbutanal, which has a chocolatey aroma, was not detected in either EP2 or WP coffee. In a previous study, it has been selected as a discriminant marker to distinguish elephant dung coffee from the controls [5]. However, in our study, a relatively similar amount of 2-methylbutanal was found in one of the elephant dung coffees (EP1) and DP coffee. High concentrations of 2-ethylpyrazine and 2-methyltetrahydrofuran-3-one (3.51 mg/L and 3.15 mg/L of brewed coffee, respectively) were found in EP2 coffee as compared to coffee obtained by other treatments. The EP1, WP, and DP coffee had 1.21, 1.37, and 1.46 mg/L of 2-ethylpyrazine in brewed coffee, respectively. The WP coffee had 2.93 mg/L of 2-methyltetrahydrofuran-3-one (Table 2). However, 2-methyltetrahydrofuran-3-one was not detected in EP1 and DP coffee. The following five compounds were found only in the EP1 coffee sample (Table 3): 2-hydroxymethylpyrrole (0.11 mg/L), 3-methylfuran (0.10 mg/L), 2-methylfuran (0.08 mg/L), 2-ethyl-3-methylpyrazine, (0.06 mg/L) and 2-hexanol (0.03 mg/L). We found four compounds to be present only in EP2 coffee. These are propionic acid (0.16 mg/L), 4-ethylguaiacol (0.15 mg/L) 1-furfurylpyrrole (0.07 mg/L), and 2-methylphenol (0.04 mg/L) (Table 3). Among them, 4-ethylguaiacol is one of the predominant volatile compounds in roasted coffee and a potent odorant in raw Arabica coffee beans but only if its concentration exceeds the odor threshold [55]. However, the presence of 4-ethylphenol, 4-ethylguaiacol, and 4-ethylcatechol spoils the flavor of red wines, and its odor is described as smoky, horsy, barnyard, and medicinal [56]. These volatile phenol compounds are products of free hydroxycinnamic acids, and their ethyl esters by the actions of *Brettanomyces/Dekkera* yeasts [56]. The compounds found only in EP1 and EP2 coffee beans may be used to discriminate the elephant dung coffee from the others, and they also allow to discriminate EP1 from EP2 coffee samples.

**Figure 8:**
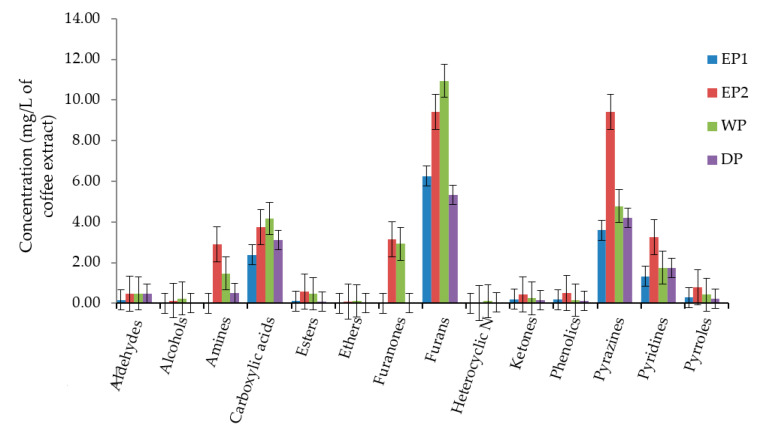
The total concentration of volatile compounds as functional classes. EP1: elephant dung coffee (pulp was completely digested); EP2: elephant dung coffee (pulp was not digested); WP: wet-processed coffee beans; DP: dry-processed coffee beans.

We have compared the total concentration of volatile compounds as a functional group in coffee beans produced by each processing method. The most abundant functional classes of volatile compounds were furans, pyrazines, and carboxylic acid in all treatments. Alcohols, aldehydes, esters, ethers, ketones, heterocyclic N, phenolics, and amines were present in lower concentrations. The total concentration of furans was 10.94, 9.43, 6.25 and 5.34 mg/L of coffee extract in WP, EP2, EP1, and DP treatments, respectively (Figure 8). The total concentration of pyrazines was higher in EP2 (9.42 mg/L) compared to EP1 (3.59 mg/L), WP (4.78 mg/L)m, and DP (4.22 mg/L). As presented in Figure 8, the carboxylic acids functional class was relatively higher in WP and EP2 coffee. In general, the total concentration of most of the functional classes of volatile compounds was lower in DP coffee as compared to others.

### 3.7. Principal Component Analysis

**Figure 9:**
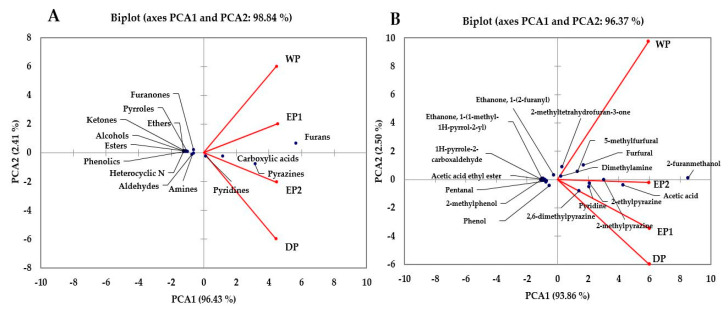
Biplot of the principal component analysis of the volatile classes (**A**) and individual compounds (**B**) of coffee obtained using different processing methods. EP1: elephant dung coffee (pulp was completely digested); EP2: elephant dung coffee (pulp was not digested); WP: wet-processed coffee beans; DP: dry-processed coffee beans.

PCA is a statistical tool developed to explain differentiation between samples and to obtain information from the variables that largely affect the spatial distribution in samples. This method can reduce the dimensionality of the data matrix while maintaining most of the information of the initial data and can also describe the relationship of objects and the correlation structure of the variables [57]. The principal component analysis of the volatile compounds was made and is presented in Figure 9A,B. The PCA was performed using the total %RPA of volatile classes: furans, pyrazines, carboxylic acid, alcohol, aldehydes, esters, ethers, ketones, heterocyclic N, pyrroles, pyridines, furanones, phenolics, and amines as variables. The results of the PCA are presented in Figure 9A. The PCA comprised about 98.84% of the total variance in two components (Figure 9A). The PCA1 accounted for about 96.43% of the variance and positively correlated to furans, pyrazines, carboxylic acids, and pyridines. The PCA2 accounted for 2.41% of the total variance. Furans were higher in EP1 and WP bean samples. According to the %RPA of the functional classes, the treatments were separated. The volatile functional classes that have been found in higher (furans, carboxylic acid, and pyrazines) or lower (phenolic, pyrroles, alcohols, and ketones) amounts in the WP treatments might be responsible for separating the WP treatments in a wide distance compared to EP2 and DP in Figure 9A. The volatile functional classes had more or less similar patterns in both elephant dung coffee samples (EP1 and EP2). The volatile classes were patterned differently between EP1 and DP, and between WP and DP, treatments. As presented in Figure 9B, PCA was carried out using the relative proportions of individual volatile compounds in brewed coffee samples. The PCAs account for 96.37% of the total variance (Figure 9B). The PCA1 accounts for 93.86% of the total variance and was positively correlated to 2-furanmethanol, acetic acid, pyridine, 2-methylpyrazine, 2,6-dimethylpyrazine, 5-Methylfurfural, furfural, dimethylamine, and 2-methyltetrahydrofuran-3-one. The PCA2 accounts for 2.50% of the total variances (Figure 9B). The WP treatment showed a clear difference based on individual volatile compounds relative percentages of peak areas. The volatile compounds that have been found in higher (acetic acid, 2-methylpyrazine, 5-methylfurfural, furfural etc.), and lower (2,6-dimethylpyrazine, Pyridine, 2-ethylpyrazine etc.) amounts in the WP treatment might be responsible for resulting this big separation of WP treatment as compared to other treatments in Figure 9B. The difference between EP1 and EP2 treatments was not as big as compared to WP. This indicates that the responses of volatile compounds in both EP1 and EP2 samples were relatively similar.

## 4. Conclusions

We have compared the antioxidant and volatile compounds of coffee obtained using different methods. To the best of our knowledge, only one experiment has been conducted to compare the volatile compounds of elephant dung coffee beans with the coffee beans from another process as control. A change in antioxidant activity, TPC, TFC, and TTC has been noted in both green and roasted beans obtained using different processing methods. The TPC, TFC, and TTC of green coffee beans were higher compared to roasted coffee beans and then decreased as the roasting durations extended from light to dark levels. In general, the elephant dung coffee samples had better results of DPPH, FRAP, TPC, TFC, TTC and volatile compounds relative to other treatments. However, the result of each parameter was not consistent at all roasting levels. Regarding the roasting conditions, light roasting could be considered the best to have higher amounts of DPPH, FRAP, TPC, TFC, and TTC in the coffee extracts. Relatively abundant amounts of 2-furanmethanol, acetic acid, 2-methylpyrazine, 2,6-dimethylpyrazine, pyridine, and 5-methylfurfural compounds were found from all coffee beans treatments. Furans and pyrazines were major volatile functional classes found in coffee beans from all treatments. These compounds have been detected only in EP1 and EP2 coffee beans. EP1 beans alone contained 2-hydroxymethylpyrrole, 3-methylfuran, 2-methylfuran, 2-ethyl-3-methylpyrazine, and 2-hexanol compounds. Propionic acid, 4-ethylguaiacol, 1-furfurylpyrrole, and 2-methylphenol were found only in the EP2 beans. These compounds need further study to differentiate the elephant dung coffees from the coffees produced by wet and dry processing methods. Further, the results from this study will serve as inputs for more comprehensive studies.

## Figures and Tables

**Table 1:** Colorimeter data of roasted coffee that obtained using different processing methods.

Roast Levels	Treatments	Color Parameters		
		L*	a*	b*
Light roasted	EP1	36.46 ± 0.50 ^b^	4.75 ± 0.23 ^a^	11.37 ± 0.52 ^b^
	EP2	38.12 ± 0.17 ^a^	5.18 ± 0.24 ^a^	12.38 ± 0.27 ^a^
	WP	34.56 ± 0.64 ^c^	5.48 ± 0.25 ^a^	11.00 ± 0.19 ^b^
	DP	33.81 ± 0.34 ^c^	5.56 ± 0.07 ^a^	10.27 ± 0.21 ^c^
Medium roasted	EP1	33.42 ± 0.47 ^b^	4.38 ± 0.05 ^b^	8.38 ± 0.33 ^c^
	EP2	35.41 ± 0.37 ^a^	4.6 ± 0.0.21 ^b^	9.55 ± 0.28 ^a^
	WP	33.81 ± 0.14 ^b^	4.07 ± 0.12 ^c^	8.04 ± 0.05 ^c^
	DP	32.19 ± 0.22 ^c^	4.97 ± 0.13 ^a^	9.00 ± 0.12 ^b^
Dark roasted	EP1	31.22 ± 0.24 ^c^	4.08 ± 0.05 ^a^	6.86 ± 0.08 ^b^
	EP2	34.40 ± 0.23 ^a^	4.20 ± 0.08 ^a^	8.60 ± 0.25 ^a^
	WP	32.79 ± 0.34 ^b^	3.23 ± 0.07 ^c^	6.14 ± 0.15 ^c^
	DP	31.41 ± 0.20 ^c^	3.84 ± 0.20 ^b^	7.00 ± 0.19 ^b^

Note: Means denoted with different letters (^a–c^) showed a significant difference at *p* < 0.05 within each column at each roasting condition. EP1: elephant dung coffee (pulp was completely digested); EP2: elephant dung coffee (pulp was not digested); WP: wet-processed coffee beans; DP: dry-processed coffee beans.

**Table 2:** Volatile compounds identified from coffee beans obtained using different processing methods (EP1, EP2, WP, and DP).

Concentration mg/L of Coffee Extract								
No.	RT	Compound	EP1	EP2	WP	DP	Odor Type	Functional Group
1	31.062	2-furanmethanol	4.76 ± 0.92	8.12 ± 1.00	7.54 ± 1.03	4.11 ± 0.79	Bready	Furans
2	26.002	Acetic acid	2.38 ± 0.34	3.60 ± 0.50	4.17 ± 0.26	3.10 ± 0.24	Acidic	Carboxylic acid
3	15.502	2-methylpyrazine	1.13 ± 0.12	3.10 ± 0.39	3.86 ± 0.57	2.74 ± 0.63	Nutty, roasted, chocolate, cocoa	Pyrazine
4	17.88	2,6-dimethylpyrazine	1.20 ± 0.64	2.83 ± 0.94	0.92 ± 0.22	1.48 ± 0.31	Nutty, sweet, fried	Pyrazine
5	12.191	Pyridine	1.33 ± 0.29	3.26 ± 1.03	1.75 ± 0.06	1.74 ± 0.65	Fishy	Pyridine
6	26.776	5-methylfurfural	1.10 ± 0.85	1.26 ± 0.04	2.48 ± 3.04	0.93 ± 0.12	Caramelly	Furans
7	4.949	2-butanone	0.13 ± 0.19	0.16 ± 0.01	0.18 ± 0.13	0.13 ± 0.07	Ethereal	Ketones
8	3.689	Acetaldehyde	0.09 ± 0.07	0.09 ± 0.01	0.13 ± 0.02	0.11 ± 0.02	Fruity	Aldehyde
9	23.082	Furfural	0.87 ± 0.14	1.31 ± 0.31	2.01 ± 0.57	1.08 ± 0.07	Bready	Furans
10	20.826	Dimethylamine	ND	2.91 ± 0.52	1.47 ± 0.09	0.51 ± 0.07	Fishy, ammonia-like	Amine
11	28.45	1-methylpyrrole-2-carboxaldehyde	0.16 ± 0.07	0.50 ± 0.11	ND	0.15 ± 0.03	-	Pyrroles
12	4.783	Acetic acid ethyl ester	0.12 ± 0.08	0.38 ± 0.02	0.15 ± 0.03	ND	Pear drops	Esters
13	14.617	Pentanal	ND	0.25 ± 0.02	0.26 ± 0.05	0.35 ± 0.05	bready, fruity, nutty	Aldehyde
14	37.057	Guaiacol	0.17 ± 0.07	0.21 ± 0.09	0.07 ± 0.01	0.10 ± 0.04	Phenolic	Phenolic
15	29.498	Ethanone, 1-(1-methyl-1H-pyrrol-2-yl)	0.02 ± 0.01	0.19 ± 0.03	0.14 ± 0.02	ND	Earthy	Pyrroles
16	27.914	Formic acid ethyl ester	ND	0.19 ± 0.04	0.32 ± 0.04	0.07 ± 0.03	Fruity	Esters
17	23.919	Benzaldehyde	0.08 ± 0.02	0.13 ± 0.07	0.09 ± 0.01	0.04 ± 0.02	Fruity	Aldehyde
18	7.828	2,3-pentanedione	ND	0.06 ± 0.01	0.07 ± 0.01	0.04 ± 0.01	Buttery, oily, caramel-like	Ketones
19	42.806	1H-pyrrole-2-carboxaldehyde	ND	0.03 ± 0.00	0.11 ± 0.03	0.06 ± 0.01	Musty, beefy, coffee	Heterocyclic N
20	41.046	2-acetylpyrrole	ND	0.03 ± 0.00	0.28 ± 0.06	0.07 ± 0.03	Musty	Pyrroles
21	24.469	Ethanone, 1-(2-furanyl)	0.21 ± 0.06	0.29 ± 0.03	0.92 ± 0.22	0.30 ± 0.06	Sweet, almondy, nutty	Furan
22	4.758	2-methylbutanal	0.52 ± 0.15	ND	ND	0.43 ± 0.08	Chocolatey	Aldehyde
23	17.646	2-ethylpyrazine	1.21 ± 0.42	3.51 ± 0.13	1..37 ± 0.46	1.46 ± 0.13	cocoa; musty; nutty	Pyrazine
24	15.07	2-methyltetrahydrofuran-3-one	ND	3.15 ± 0.15	2.93 ± 0.62	ND	Sweet	Furanone
25	6.083	2-pentanone	0.07 ± 0.03	0.22 ± 0.06	0.07 ± 0.02	ND	fruity	Ketones
26	23.624	1-propanol, 2-methyl	ND	0.13 ± 0.03	0.24 ± 0.04	ND	Ethereal	Alcohol
27	40.503	Difurfuryl ether	ND	0.08 ± 0.01	0.12 ± 0.02	ND	coffee	Ether
28	42.266	Phenol	ND	0.05 ± 0.01	0.15 ± 0.02	ND	Phenolic, plastic	Phenolic

Note; RT: retention time; EP1: elephant dung coffee (pulp was completely digested); EP2: elephant dung coffee (pulp was not digested); WP: wet-processed coffee beans; DP: dry-processed coffee beans; RPA%: percentage of relative peak area. ND: not detected

**Table 3:** Volatile compounds found only in EP1 and EP2 brewed coffee samples.

EP1 Coffee Sample							
No.	RT	Compound	Area	RPA%	Concentration (mg/L of Brewed Coffee)	Odor Type	Functional Group
1	13.089	2-hydroxymethylpyrrole	3092797	0.24 ± 0.04	0.11 ± 0.02	-	Pyrrole
2	4.103	3-methylfuran	2861634	0.25 ± 0.01	0.10 ± 0.01	-	Furan
3	4.254	2-methylfuran	2357722	0.18 ± 0.03	0.08 ± 0.01	Chocolatey	Furan
4	20.184	2-ethyl-3-methylpyrazine	1764823	0.145 ± 0.02	0.06 ± 0.00	Nutty	Pyrazine
5	5.113	2-hexanol	693878	0.05 ± 0.01	0.03 ± 0.00	Earthy	Alcohol
**EP2 Coffee Sample**							
6	28.165	Propionic acid	4715661	0.14 ± 0.02	0.16 ± 0.02	Pungent, rancid, unpleasant	Carboxylic acid
7	42.46	4-ethylguaiacol	4271764	0.13 ± 0.05	0.15 ± 0.02	Spicy	Phenolic
8	34.812	1-furfurylpyrrole	2088675	0.06 ± 0.01	0.07 ± 0.01	Vegetable	Pyrrole
9	41.886	2-methylphenol	1120466	0.03 ± 0.01	0.04 ± 0.00	sweet, phenolic	Phenolic

Note; RT: retention time; EP1: elephant dung coffee (pulp was completely digested); EP2: elephant dung coffee (pulp was not digested); RPA%: percentage of relative peak area.
